# Endograft Sizing for Endovascular Aortic Repair and Incidence of Endoleak Type 1A

**DOI:** 10.1371/journal.pone.0158042

**Published:** 2016-06-30

**Authors:** Ruben V. C. Buijs, Clark J. Zeebregts, Tineke P. Willems, Tryfon Vainas, Ignace F. J. Tielliu

**Affiliations:** 1 Department of Surgery, Division of Vascular Surgery, University Medical Center Groningen, Groningen, The Netherlands; 2 Department of Radiology, University Medical Center Groningen, Groningen, The Netherlands; University Francisco de Vitoria School of Medicine, SPAIN

## Abstract

**Objective:**

In endovascular aortic aneurysm repair (EVAR), proximal type 1A endoleaks can occur as a result of hostile neck anatomy or over- or undersizing of the endograft. As the current standard is based on the diameter or average of the short and long axes in a central lumen reconstruction image, it can falter in irregularly shaped aortic necks. An alternative method is circumference-based, therefore minimizing the measurement error. In this study we aimed to assess the degree of discrepancy between both methods and the association of this discrepancy with the occurrence of endoleak type 1A.

**Methods:**

All patients with early (<30 days post-operative) endoleak type 1A after elective EVAR at our center between 2004 and 2016 were identified for a retrospective case-control study. Control patients were matched based on hostile neck anatomy, such as calcification, thrombus, reverse taper, and β-angulation. The aortic neck diameter was measured using the traditional, diameter-based method as well as an alternative method, based on the circumference of the aortic neck.

**Results:**

In 482 EVAR patients, 18 early endoleak type 1A cases were found (3.9%). After exclusion, 12 cases remained and 48 matching controls were found. No significant differences were found between the two measuring methods at any level below the renal arteries. The inter-observer variability was significant for the D(mean) (0.4 ± 1.69 mm, P = .02) and was larger than the D(circ) method (-0.1 ± 1.03 mm, P = .35). In only four out of 12 cases the endograft size was 10–20% larger than the D(mean) and D(circ) measurements. The differences between the diameter of the D(mean) and D(circ) and the chosen endograft were smaller for the case group (-8 ± 25.6% and -7 ± 24%) than for the control group. (-12.4 ± 12.4% and -11 ± 10.7%).

**Conclusion:**

The difference between the D(mean) and D(circ) methods for aortic neck measurement was not large enough to play a significant role in the incidence of endoleak type 1A. Inadequate oversizing and considerable β-angulation of the aortic neck may have been the cause of endoleak type 1A in this population. Robust and well-investigated sizing methods are paramount for accurate endograft sizing and prevention of endoleak type 1A. Therefore the lack of studies in this field and a sizeable inter-observer variability do not justify the widespread reliance on the traditional diameter-based methods for endograft sizing.

## Introduction

The introduction of endovascular techniques for the treatment of abdominal aortic aneurysms (AAA) has lead to a decrease in perioperative mortality. With regards to long-term postoperative mortality, endovascular aortic repair (EVAR) is on par with open repair [1,2]. However, in terms of complications patients treated with EVAR have a three- to fourfold higher rate of graft-related morbidity than patients treated by open repair [3].

A large part of EVAR-related complications is due to endoleaks. In the case of endoleak type 1A, the endograft does not completely seal the proximal aneurysm neck and arterial flow is present between the wall of the aortic neck and the graft material. This flow may lead to further growth and eventually rupture of the aneurysm. The incidence of endoleak type 1 can be partially attributed to the surgical skill of the surgeon, as well as to the skill for preoperative sizing of the endograft [4]. Hostile neck characteristics have also been associated with the occurrence of endoleak type 1A. These characteristics include a short proximal aneurysm neck length, reverse tapering of the neck, mural calcification or thrombus, and severe neck angulation [5].

Endografts are currently sized by determining the diameter of the aortic neck. This is measured by averaging the diameter of the longest and shortest axis of the infrarenal aortic neck. Since the introduction of EVAR, this has been the only method to size endografts. As the diameter-based method has always been considered to be the best and no comparative studies have been performed so far, it is conceivable that there are other, more accurate methods available. In cases where the central lumen line of the aortic neck section is not a perfect circle, the abovementioned method will yield some mathematical incorrectness. Recently, a different and mathematically more correct measurement of the diameter of the aortic neck, based on the circumference of the neck, has been proposed. Theoretically, both methods produce similar results as long as the section of the aortic neck has a perfect circular shape. However, as aortic neck cannot be perfectly circular, there will always remain a discrepancy between the results of the two methods [6]. Still, the impact of this discrepancy for potential clinical use of both methods is unclear. Also, most endograft manufacturers recommend that the diameter of the endograft should be 10–20% larger than the diameter of the aortic neck, also known as “oversizing”. In a systematic review, Van Prehn et al. evaluated risks and benefits of oversizing [7]. The authors concluded that the evidence for a correlation between oversizing and incidence of endoleak type 1A is limited, but oversizing between 10–20% is recommended and “relatively safe”. Theoretically, adequate oversizing should negate any clinical consequences of the discrepancy between the current method and its alternative, but this has never been studied. The aim of our study was to assess the degree of discrepancy between both methods and the association of this discrepancy with the occurrence of endoleak type 1A. To this end, the traditional diameter-based method and the alternative circumference-based method were compared in a retrospective, case-control set-up.

## Methods

### Study Design

The aim of this study was to assess the discrepancies between both methods and the association of this discrepancy with the occurrence of endoleak type 1A in a retrospective case-control set-up. Two methods for sizing the aortic neck diameter were retrospectively applied to an endoleak type 1A (case) group and a matched control group. For both case and control subjects, demographic and clinical data were gathered. These included sex, age, smoking habits, history of cardiovascular disease and/or diabetes mellitus type 1 or 2, peri- and postoperative complications, peri- and postoperative treatment of endoleak type 1A, death following intervention, and aneurysm morphology. For additional information on the endografts that were chosen as part of routine clinical care, the manufacturer brand and endograft size were collected. For this project, the Medisch Ethische Toetsingscommissie (Medical Ethics Committee) of the University Medical Center Groningen approved this study (number 201500390) and decided that no informed consent was required.

### Patients

All subjects were collected from a database of AAA patients who underwent elective EVAR treatment between January 2004 and January 2016. Case subjects with an endoleak type 1A that were identified by a vascular surgeon during or early after (<30 days) EVAR treatment, were included in the study. Late endoleak type 1A cases were excluded, as these cases are commonly a result of ongoing aneurysmal disease. The inclusion criteria for case subject eligibility were as follows: 1. Identification of an endoleak type 1A diagnosed intraoperatively or on the first computed tomography angiogram (CTA), routinely performed four weeks postoperatively; 2. The availability of CTA data. Case and control subjects that had undergone EVAR treatment for impending or acute aneurysm rupture were excluded from this study. Patients who received custom made endografts because of severe anatomic restrictions were also excluded.

Control subjects were EVAR-treated patients with no post-operative complications, and were matched to the cases based on hostile neck anatomy characteristics.^4^ Matching was performed to correct for the previously described association of these confounding factors with the occurrence of endoleak type 1A. Hostile neck anatomy was defined as having at least one of the following anatomical characteristics of the aortic neck: length <10 mm, tapering >2 mm per 10 mm of length, diameter >28 mm, >50% neck circumference lined with thrombus, >50% neck circumference lined with calcification, and the β-angle between neck and aneurysm of >60 degrees [8–13]. Each case subject was paired with control subjects based on their configuration of hostile neck anatomy characteristics. Because of the low incidence of endoleak type 1A, a ratio of four controls for each case was chosen to maximize the statistical power [14].

### Computational Aortic Neck Measurements

**Fig 1:**
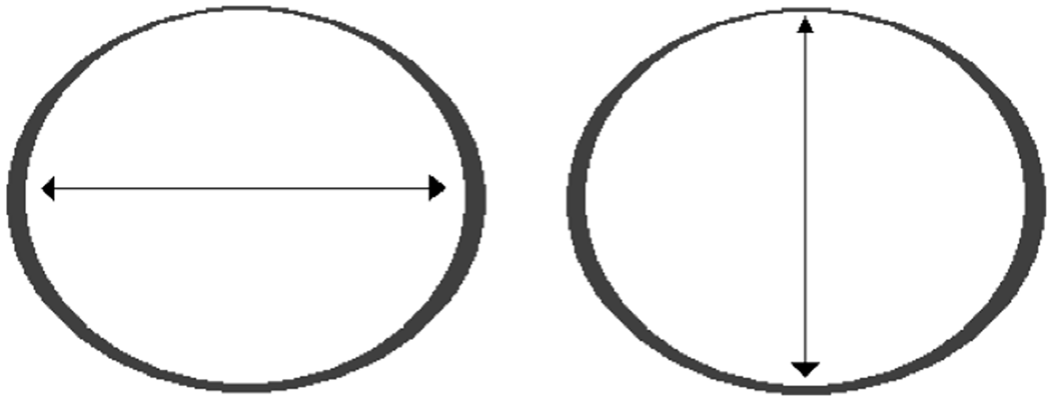
Aortic neck diameter assessment method according to the current standard. The average of long (left) and short (right) axis provide the diameter of the corresponding circle.

The aortic neck diameter was measured with two methods. Mathematical equations and definitions are explained to provide full understanding of the study design. Sizing of endografts is generally performed by measuring the diameter (D) of the aortic neck (Fig 1). In cases where the axial plane of the neck is not a perfect circle, the average of long and short axes are used to calculate the diameter of the corresponding circle:

D(mean)=(long axis+short axis)÷2 (1)

In theory, the circumference (C) of the aortic neck can also be used to calculate the diameter of the corresponding circle:

$$D\left(circ\right)=\frac{C}{\pi }$$ (2)

**Fig 2:**
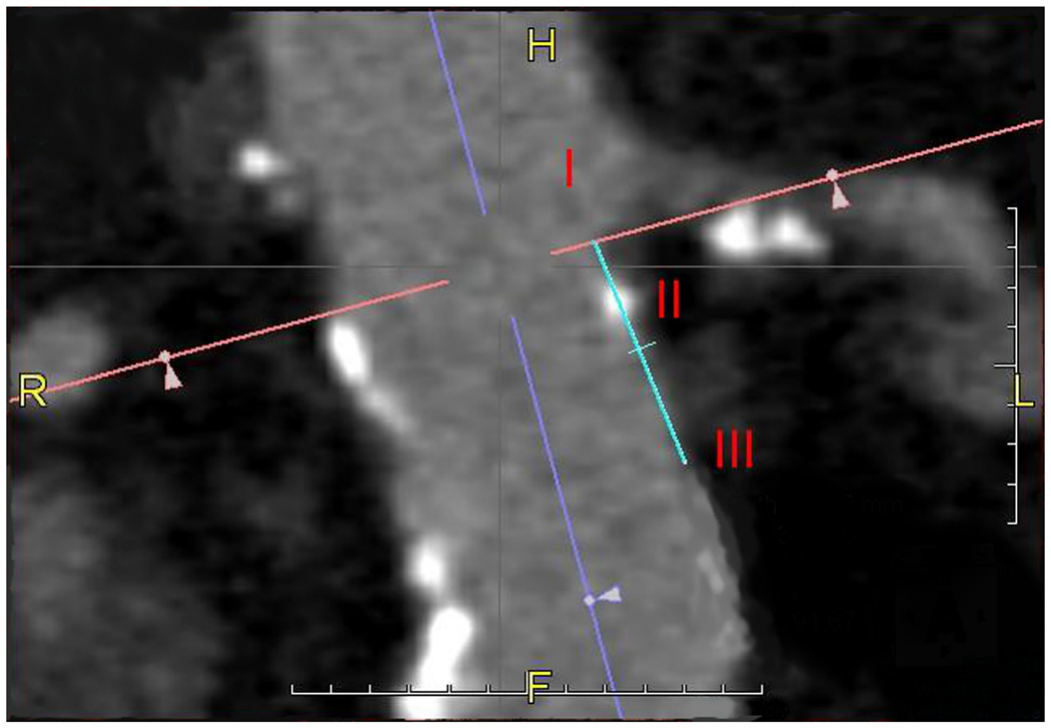
Computed tomography image of the abdominal aorta. In Roman numerals the infra renal distances are portrayed. I: 0 mm, II: 7.5 mm, and III: 15 mm. To provide a standardized method for measuring the distances as mentioned above, first the midline of the aortic neck is chosen. The perpendicular line is then placed at the point where the lowest renal artery branches from the aorta. In the caudal direction from this point (I) and parallel to the midline, a distance of 7.5 and of 15 mm is measured to identify point II and III, respectively.

Both the D(mean) and D(circ) were determined at three levels of the aortic neck: at the level just below the distal border of the most distal renal artery (0 mm, height I), at 7.5 mm (height II), and 15 mm (height III) below this level. (Fig 2) At each of these locations, relevant sections were selected based on the center lumen line. The D(circ) method was performed by manually tracing the aortic wall using the software-specific digital calipers. The degree of thrombus, calcification, tapering, and angulation were measured as described in earlier reports [8,10–12]. Two observers, including the main investigator (first author) and an experienced (endo)vascular surgeon (last author) were instructed on how to perform both methods and were blinded to the groups. Blinding was achieved through randomizing the order of the subjects in the database before measuring. The results of the D(mean) and D(circ) methods obtained by the main investigator and the expert were compared to evaluate the inter-rater reliability of both methods. Differences between observers, methods, and between case and control groups were considered clinically relevant in case they exceeded 10%, based on the fact that this is considered the lower threshold for adequate oversizing [7].

**Fig 3:**
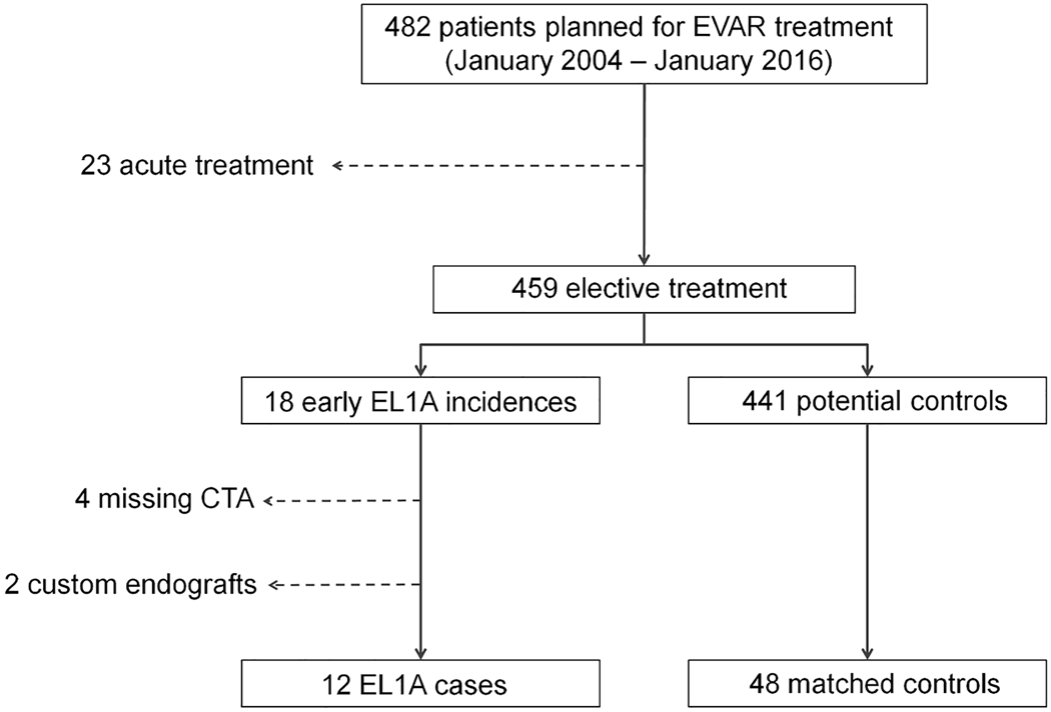
Population selection in this study. Of the 482 patients with AAA who were selected for EVAR treatment, 23 received acute intervention and were excluded. EL1A was found in 18 patients, two of which received custom made endografts and in four cases the CTA images were missing. These were therefore excluded. Of the 441 remaining EVAR, 48 controls were selected based on matching for hostile neck anatomy. AAA: abdominal aortic aneurysm. EVAR: endovascular aneurysm repair. EL1A: endoleak type 1A. CTA: computed tomography angiography.

To enable case matching with controls, all subject CTA data were analyzed using AquariusNet Viewer Client V4.4.4.23 (TeraRecon, Foster City, CA, USA) on a 24-inch NEC MultiSync EA241WM monitor. This was performed orthogonally to a center line using multi planar reformat views (Fig 3). The neck length was measured computationally as the distance between the distal border of the lowest renal artery and the proximal start of the AAA. An angulation measurement method based on the method by van Keulen et al. was performed using a manually adjusted estimation of the central lumen line [12]. The angle between the anticipated landing zone of the endoprosthesis and the aneurysm sack (β angle), was measured. Calcification was identified as high attenuating signals (>140 Hounsfield units) within, or closely aligned with, the vessel wall. It was then graded as either more or less than 50% circumferential calcification at the anticipated landing zone [4]. Low attenuating signals lining the lumen of the vessel wall were identified as thrombus. Thrombus was graded as either more or less than 50% circumferential thrombus with a thickness of >2 mm [12]. Reverse tapering was defined as an increase in neck diameter of >2 mm over a length of 10 mm [10].

### Statistical Analysis

The sample size of this study was chosen based on the incidence of early endoleak type 1A in our EVAR-treated patient population, following the above-mentioned inclusion and exclusion criteria. A post-hoc power analysis was performed using G*Power 3.1.9.2 for Mac OS X (University of Düsseldorf, Düsseldorf, Germany) to assess whether the group sizes were adequate to reliably calculate the degree of significance [15]. The primary endpoint for this study was the difference in diameter (mm) between D(mean) and D(circ). Categorical data were tested in cross-tabs using the chi-square test and Mantel-Haenszel matched-pairs analysis. Continuous data were analyzed using paired t-tests if normally distributed. In case of skewed distribution of data, the Wilcoxon signed rank sum test was used. Inter-observer variability and the variability between the D(mean) and D(circ) method were calculated through paired-samples t-tests and visualized in Bland-Altman plots. Results were reported as a percentage for categorical data, mean ± standard deviation (SD) for continuous data, and median and interquartile range in case the data had a skewed distribution. Significance was set at p < .05. Statistical analyses were performed using SPSS 20 (Statistical Package for the Social Sciences, IBM, Armonk, NY, USA).

#### Post-hoc power analysis

To calculate the effect size (*d*), means and standard deviations of the D(mean) measurements were used, providing a *d* of .47. Given the sample sizes of both the case and control group and an α of .05, the post-hoc power was calculated to be at .3. As the minimally acceptable power is set at .8, the statistical significance of potential differences is not representative of the general population. All the diameters from height I to III were compared for the inter-observer variability and the paired-samples t-test for the variability between the D(circ) and D(mean) method. These could be reliably tested for significance, as the number of unique diameter measurements was tripled.

## Results

### Demographics and Clinical Information

**Fig 4:**
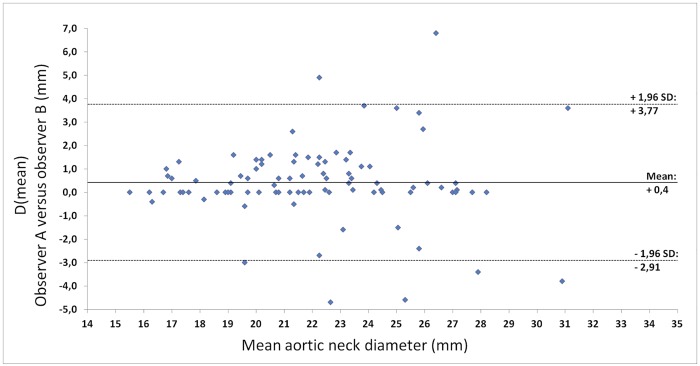
Bland-Altman plot of the differences between observer A and B for the D(mean) method.

**Fig 5:**
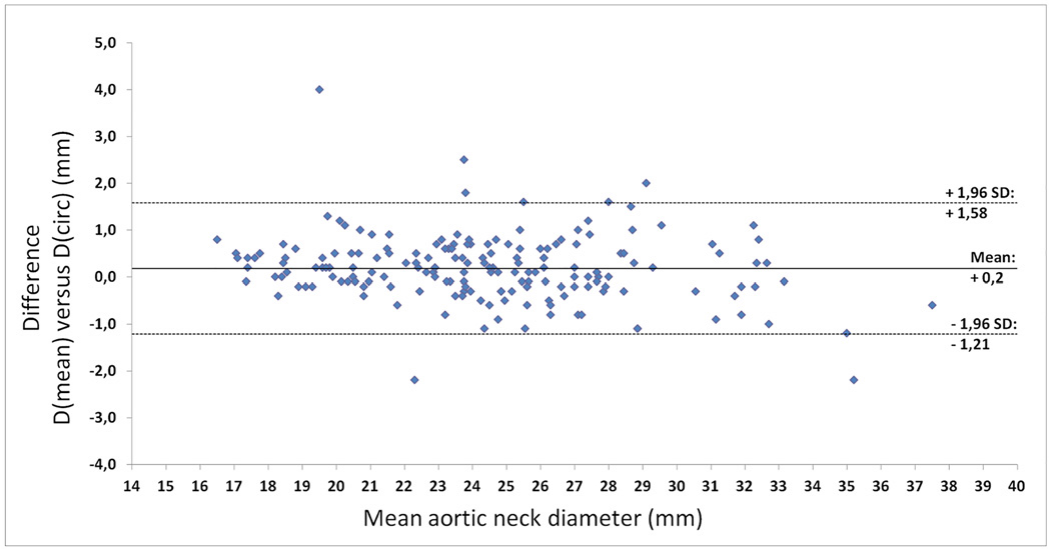
Bland-Altman plot of the differences between D(mean) and D(circ) measurements of both observers.

Comparison of endoleak type 1A patients to controls is only reliable if the differences in demographic and clinical variables are small. Patients were stratified based on incidence of endoleak type 1A, and clinical data. Twenty-three out of 482 patients planned for elective EVAR underwent acute treatment for a ruptured or symptomatic aneurysm and were excluded from the study. Of the 459 remaining elective patients, 18 (3.9%) were diagnosed with an early endoleak type 1A. In four patients CTA data were missing and two patients had received custom made endografts, leaving 12 endoleak type 1A patients for CTA analysis. After matching for hostile neck anatomy, 48 patients without complications were found as control cases (Fig 3). Minor differences between both groups were found in age, sex, and cardiovascular history. Most notable was the fact that the incidence of diabetes mellitus type 1 or 2 was 10% higher in the control group than in the case group. The maximum diameters of the AAA did not differ significantly between the two groups (Table 1). Inter-observer variability was significant for the D(mean) (0.4 ± 1.69 mm, P = .02) and larger than for the D(circ) method (-0.1 ± 1.03 mm, P = .35) (Figs 4 and 5). The greatest part of the observations for both methods is within the 95% limits of agreement. However, the 95% limit spans 3.37 mm for the D(mean) method or a 14% difference to the average D(mean) measured in this population. For the D(circ) method the 95% limit spans 2.05 mm, which is an 8.1% difference to the average D(circ) measured in this population.

**Table 1:** Demographic characteristics of case and control groups.

Variable	Endoleak type 1A (n = 12)	Controls (n = 48)
Age (y)	75.8 ± 11.9	71.8 ± 7.7
Male sex (%)	75	89.6
History of cardiovascular disease (%)	75	72.9
History of diabetes mellitus type 1 or 2 (%)	8.3	18.8
AAA diameter (mm)	64.4 ± 14.8	62.7 ± 13.2
Graft (Brand/Type)		
Gore/Excluder (%)	25	41.7
Cook/Zenith (%)	50	50
Vascutek/Anaconda (%)	25	8.3
Compliance to the Instructions For Use (%)	83.3	79.2

AAA: abdominal aortic aneurysm

y: years

### Characteristics of Endoleak Type 1A Patients

Clinical, anatomical, and graft-related characteristics at individual level can be valuable in assessing the risk to develop endoleak type 1A. The hostile neck characteristics and treatment of all endoleak type 1A patients are shown in Table 2. Nine patients (75%) had ≥40° β-angulation of the aortic neck and for three patients (25%) this was ≥60°. Three others had a >50% calcified aortic neck circumference. No other hostile neck anatomy characteristics were found. Although the annual endoleak type 1A incidence in this population is spread out evenly between 2004 and 2012, three cases were found in 2013 alone and none from 2014 to 2016. In two cases, the endograft required additional ballooning for the endoleak to diminish, but in none of the cases the endoleak had disappeared perioperatively. With and without ballooning, watchful waiting proved adequate on the first CTA two months after the intervention in 10 out of 12 cases. For two patients, an additional intervention was needed. In one case the endoleak type 1A was treated by open surgery and aortic banding and in the other case the endograft was extended proximally with a fenestrated cuff. In both cases the endoleak had disappeared after the second intervention.

**Table 2:** Characteristics and treatment of endoleak type 1A in the case group.

EL1A case	Year	Brand	Neck angle	≥50% thrombus	≥50% calcified	≤10 mm length	Reverse taper	>28 mm neck diameter	EL1A Treatment
1	2004	Cook	50°	No	Yes	No	No	No	WW
2	2006	Cook	80°	No	No	No	No	No	WW
3	2007	Cook	50°	No	Yes	No	No	No	Aortic banding
4	2008	Cook	45°	No	No	No	No	No	Ballooning + WW
5	2009	Cook	100°	No	No	No	No	No	Ballooning + WW
6	2009	Cook	30°	No	No	No	No	No	WW
7	2010	Vascutek	90°	No	No	No	No	No	WW
8	2011	Vascutek	55°	No	No	No	No	No	WW
9	2012	Gore	40°	No	Yes	No	No	No	WW
10	2013	Gore	35°	No	No	No	No	No	Fen. cuff ext.
11	2013	Vascutek	55°	No	No	No	No	No	WW
12	2013	Gore	20°	No	No	No	No	No	WW

EL1A: endoleak type 1A

WW: watchful waiting

Ballooning: extra ballooning of the endograft in the aortic neck

Fen. cuff ext.: extension of the endograft using a fenestrated cuff

Table 3 shows that the D(mean) and D(circ) measurements differed by -3.1 to 5.3% from each other. In comparison to the endograft diameter, the D(mean) measurements were between 13.7% larger to 16.8% smaller. D(circ) measurements were between 12.4% larger to 13.8% smaller than the oversized endografts. In only four out of 12 cases the endograft size was 10–20% larger than the D(mean) and D(circ) measurements.

**Table 3:** Case group neck measurements, endograft size, and comparisons.

EL1A case	D(mean) average (mm)	D(circ) average (mm)	Diff. D(mean)-D(circ) (%)	Endograft diameter (mm)	Diff. Graft-D(mean) (%)	Diff. Graft-D(circ) (%)
1	26.5	27.9	5.3	26	1.9	6.8
2	29.0	28.9	-0.3	26	10.3	10.0
3	25.5	25.4	-0.4	24	5.9	5.5
4	27.8	27.4	-1.4	24	13.7	12.4
5	25.7	25.6	-0.4	24	6.6	6.3
6	29.7	30.5	2.7	30	-1.0	1.6
7	24.0	24.6	2.5	28	-16.7	-13.8
8	32.7	31.7	-3.1	34	-4.0	-7.3
9	19.7	20.4	3.6	23	-16.8	-12.8
10	25.2	25.7	2	28	10	8.2
11	20.9	20.9	0	30	30	30
12	21.6	22.1	2.3	23	6.1	3.9

Diff: difference

EL1A: endoleak type 1A

### Differences between Abdominal Aortic Neck Measurements Using D(mean) versus D(circ)

The D(mean) and the D(circ) measurements were compared first. The diameters measured by D(mean) (25.0 ± 4.1 mm) were on average 1.2% smaller than those of the D(circ) method (25.3 ± 3.9 mm). The aortic neck diameters at the different levels (0 mm, 7.5 mm, and 15 mm below the lowest renal artery) were slightly larger in the case group, although not significantly (Table 4). The differences between the two methods were tabulated for both the case and control group (Table 5). The difference between the two methods was small and was not consistent among the three distances below the renal arteries. The differences between the diameter of both the D(mean) and D(circ) and the chosen endograft were smaller for the case group (-8 ± 25.6% and -7 ± 24%) than for the control group. (-12.4 ± 12.4% and -11 ± 10.7%). Although on average the 10–20% oversizing rule was upheld for the control group, the average percentage of oversizing was inadequate for the case groups.

**Table 4:** Comparison of D(mean) and D(circ) measurements and endograft size.

Variable	Endoleak type 1A (n = 12)	Controls (n = 48)
D(mean) (mm)		
0 mm infrarenal diameter	25 ± 5	23.5 ± 3.8
7.5 mm infrarenal diameter	25.5 ± 4.1	23.9 ± 4.1
15 mm infrarenal diameter	26.6 ± 4.3	24.4 ± 4.4
Mean diameter	25.7 ± 3.8	23.9 ± 3.8
D(circ) (mm)		
0 mm infrarenal diameter	25.5 ± 4.6	23.7 ± 3.8
7.5 mm infrarenal diameter	25.7 ± 4	24 ± 4
15 mm infrarenal diameter	26.6 ± 3.9	24.8 ± 4.0
Mean diameter	26.9 ± 3.4	24.8 ± 4.1
Endograft diameter (mm)	27.1 ± 3	26.6 ± 3.1

**Table 5:** Differences between methods and endograft size in case versus control groups.

Variable	Endoleak type 1A (n = 12)	Controls (n = 48)
Difference of D(mean) and D(circ) (%)		
0 mm infrarenal diameter	-2.3 ± 4.5	-0.9 ± 2.3
7.5 mm infrarenal diameter	-1.0 ± 2	-0.6 ± 2
15 mm infrarenal diameter	-.2 ± 3.5	-2.4 ± 8.5
Difference of endograft size and D(mean) (%)	-8 ± 25.6	-12.4 ± 12.4
Difference of endograft size and D(circ) (%)	-7 ± 24	-11 ± 10.7

## Discussion

This study aimed to investigate the degree of discrepancy between the traditional endograft sizing method versus an alternative method and to potentially associate this discrepancy with endoleak type 1A incidences. Although preoperative sizing of the aortic neck anatomy has become a routine task in the field of vascular surgery, little research has been done on how this should be performed optimally. Most companies that manufacture endografts for EVAR state that adequate sizing is the responsibility of the physician [16]. Some companies provide more detailed guidelines on how to perform diameter measurements, without providing evidence supporting the technique that should be used [17]. As a consequence, the skills of the clinician performing the measurements might influence the incidence of endoleak type 1A. To our knowledge, no studies have been performed regarding the method of preoperative sizing for EVAR patients. Many studies have been performed in the field of oversizing, though none have gone back to the measuring methodology [7].

Our data have shown that D(mean) and D(circ) yielded similar results. Not only between the case and control group, but also when comparing both methods. Consequently, using either of these methods for diameter measurements is not likely to result in clinically relevant differences. However, in the case group the endografts were inadequately oversized in 10 out of 12 patients. This corroborates earlier studies on oversizing decisions for endografts. As published by Van Prehn et al, oversizing of 10–20% should lead to a minimum of graft migration, folding, or endoleak [7]. Oversizing increases the radial force of the endograft, thus tightening the seal between graft and aortic wall, and alleviates the effects of the elliptic shape on erroneous diameter estimation. The D(mean) of an ellipse always underestimates the true diameter of the corresponding circle with the same circumference [6]. This study shows that this underestimation was practically not relevant, so adequate oversizing should be able to correct for this. On the other hand, aggressive oversizing can also be associated with complications, such as endoleak due to fabric pleats. This could have been the case for one endoleak type 1A patient in this population, whose endograft was oversized by 30%. Thus, it remains unclear what “adequate” oversizing is. Since it is up to the surgeon to choose what exact percentage in the 10–20% oversizing range should be taken for the endograft, there is sizeable potential for error. Remarkably, most of the endoleak type 1A cases received endografts with smaller diameters than both the D(mean) and D(circ) provided in this study.

Previously described incidences of endoleak type 1A ranged from 7.5 to 10.5%, although incidences as low as 0% and as high as 30% have been reported [5,18,19]. This shows that despite the rarity of endoleak type 1A, there is great variation in incidence between studies. The fact that the incidence of endoleak type 1A in our centre was lower than that reported in previous studies could be explained in several ways. During the entire study period, fenestrated grafts have been used to treat short neck AAA. The lower incidence of EL1A could therefore have been the result of a careful consideration of choosing fenestrated grafts or open repair instead of standard infrarenal EVAR in case of suboptimal aortic anatomy.

There were several notable results and trends that might have shaped this endoleak type 1A population. Most importantly, all case patients’ aortic necks had medium to severe β-angulation, some of which were over 90 degrees, but most over 40 degrees. Instructions for use (IFU) for most endografts generally consider a β-angulation of over 60 degrees as less favourable and therefore a contraindication [4,10,20]. This degree of angulation is part of the hostile neck anatomy characteristics and has been proven to increase the risk of endoleak type 1A. Therefore, severe angulation could partly explain why a proportion of the study population developed endoleak type 1A. However, the control group was matched on all hostile neck anatomy characteristics, thus angulation could only have made a minor contribution to endoleak type 1A risk in this population. Final noteworthy aspects to this population are the treatment strategy and the resulting success. Watchful waiting for most cases and additional ballooning of the neck in two cases were sufficient to treat the endoleak. These outcomes seem particularly benign when contrasted to previous studies. Previous risk assessments of endoleak type 1A described aneurysm growth as a result of persisting endoleak type 1A, therefore increasing the risk of post-EVAR aneurysm rupture [3,21].

This is the first study to compare the current standard for aortic neck measurement with an alternative method. Two studies investigated several approaches to the pre-operative sizing of an endograft for transcatheter aortic valve replacement. Although neither study proposed that the D(circ) method is optimal, both concluded that further studying of different sizing methods is warranted because of the variety in shapes of endograft placement sites [22,23]. Since the D(circ) method is mathematically more accurate than the traditional D(mean) method, it would make sense to further study its role in clinical practice. For prospective studies, our results should help to assess an adequate population size and period of time to maintain a power of at least 0.80. To reach the required number of EL1A cases, assuming a similar incidence, EVAR treated patients of at least three high volume vascular surgery departments should be prospectively followed for a period of 11 years.

The influence of observer bias in endograft sizing should not be underestimated. The D(mean) method was found to have a sizeable discrepancy between observers, potentially skewing the measurements by 14%. As this is well beyond the lower limit of safe oversizing, there is a considerable risk that inter-observer variability alone could lead to undersizing or too aggressive oversizing in some patients. This discrepancy was even seen despite the fact that measurements were performed in a highly controlled standardized setting, with consistent use of the central lumen line. It could therefore be expected that this inter-observer variability will be more pronounced between different surgical teams and under routine clinical circumstances. It might be possible to diminish this variability if the aortic neck diameter were to be automatically calculated by the applied measurement software. Kaladji et al. have studied a three-dimensional sizing software (Endosize) based on CTA images, and concluded that it may be as reliable as the current standard [24]. Regrettably, this is only one tool that has not been clinically validated yet, so decreasing inter-observer variability through widespread use of an automated endograft sizing tool may not happen for quite some time.

Several limitations to this study should be addressed. The retrospective approach was chosen after evaluation of its potential and limitations. Retrospective studies are prone to be affected by loss of information and selection bias. In this case, detailed information on the original endograft sizing measurements and technical reasoning of the respective surgeons in the preoperative phase were missing. However, retrospective studies are efficient and known to be more useful for phenomena with a very low incidence, such as endoleak type 1A. Selection bias was concomitantly minimized by matching for demographic, clinical, and known risk factors for endoleak type 1A. Observer bias too was minimized by blinding the observers to the patient groups. Another limitation is the fact that of the 18 patients with an endoleak, 22% were excluded because CTA data were missing. The fact that technical issues would diminish the case-population by this extent was unforeseen but irreparable. As our population of endoleak type 1A was too small considering the power analysis, statistical significance should be scrutinized and cannot be interpreted as a good representation of the relevant population. Nonetheless, to increase the clinical relevance of the descriptive data in this study, the power of the statistical analyses was increased to the fullest possible extent within the limited number of cases by matching with a 1:4 ratio [14].

## Conclusion

In summary, the difference between the D(mean) and D(circ) methods for aortic neck measurement was not large enough to have played a significant role in the incidence of endoleak type 1A. The data provided by this study potentiate further validation of the current standards in a prospective cohort. Inadequate oversizing, in combination with considerable β-angulation of the aortic neck may have been the cause of endoleak type 1A in this population. A low incidence of endoleak type 1A and a benign post-operative course were found. Robust and well-investigated sizing methods are paramount for accurate endograft sizing and prevention of endoleak type 1A. Therefore the lack of studies in this field and a sizeable inter-observer variability do not completely justify the widespread reliance on the traditional diameter-based methods for endograft sizing.

## Supporting Information

S1 File Study database. This file contains the compiled data whereupon our findings are based.
(SAV)

## References

[1] The United Kingdom EVAR Trial Investigators. Endovascular versus open repair of abdominal aortic aneurysm. N Engl J Med. 2010;362:1863–71. 10.1056/NEJMoa0909305 20382983

[2] Baas AF, Janssen KJM, Prinssen M, Buskens E, Blankensteijn JD. The Glasgow Aneurysm Score as a tool to predict 30-day and 2-year mortality in the patients from the Dutch Randomized Endovascular Aneurysm Management trial. J Vasc Surg. 2008;47:277–81. 10.1016/j.jvs.2007.10.018 18241749

[3] Schermerhorn ML, O'Malley AJ, Jhaveri A, Cotterill P, Pomposelli F, Landon BE. Endovascular versus open repair of abdominal aortic aneurysms in the Medicare population. N Engl J Med. 2008;358:464–74. 10.1056/NEJMoa0707348 18234751

[4] Veith FJ, Baum RA, Ohki T, Amor M, Adiseshiah M, Blankensteijn JD, et al Nature and significance of endoleaks and endotension: summary of opinions expressed at an international conference. J Vasc Surg. 2002;35:1029–35. 12021724 10.1067/mva.2002.123095

[5] Aburahma AF, Campbell JE, Mousa AY, Hass SM, Stone PA, Jain A, et al Clinical outcomes for hostile versus favorable aortic neck anatomy in endovascular aortic aneurysm repair using modular devices. J Vasc Surg. 2011;54:13–21. 10.1016/j.jvs.2010.12.010 21324631

[6] Tielliu IFJ, Buijs RVC, Greuter M, Vainas T, Wallis de Vries BM, Prins TR, et al Circumference as an alternative for diameter measurement in endovascular aneurysm repair. Med Hypoth. 2015;85:230–3. 10.1016/j.mehy.2015.05.004 26001992

[7] Van Prehn J, Schlösser FJ V, Muhs BE, Verhagen HJ, Moll FL, van Herwaarden JA, et al Oversizing of aortic stent grafts for abdominal aneurysm repair: a systematic review of the benefits and risks. European journal of vascular and endovascular surgery. Eur J Vasc Endovasc Surg. 2009;38:42–53. 10.1016/j.ejvs.2009.03.025 19428273

[8] Van Keulen JW, Moll FL, Barwegen GK, Vonken EP, van Herwaarden JA. Pulsatile distension of the proximal aneurysm neck is larger in patients with stent graft migration. European journal of vascular and endovascular surgery. Eur J Vasc Endovasc Surg. 2010;40:326–31. 10.1016/j.ejvs.2010.05.009 20561803

[9] Dillavou ED, Muluk SC, Rhee RY, Tzeng E, Woody JD, Gupta N, et al Does hostile neck anatomy preclude successful endovascular aortic aneurysm repair? J Vasc Surg. 2003;38:657–63. 14560209 10.1016/s0741-5214(03)00738-9

[10] AbuRahma AF, Campbell J, Stone P, Nanjundappa A, Jain A, Dean LS, et al The correlation of aortic neck length to early and late outcomes in endovascular aneurysm repair patients. J Vasc Surg. 2009;50:738–48. 10.1016/j.jvs.2009.04.061 19595545

[11] Sternbergh WC, Carter G, York JW, Yoselevitz M, Money SR. Aortic neck angulation predicts adverse outcome with endovascular abdominal aortic aneurysm repair. J Vasc Surg. 2002;35:482–6. 11877695 10.1067/mva.2002.119506

[12] Van Keulen JW, Moll FL, Tolenaar JL, Verhagen HJ, van Herwaarden JA. Validation of a new standardized method to measure proximal aneurysm neck angulation. J Vasc Surg. 2010;51:821–8. 10.1016/j.jvs.2009.10.114 20347677

[13] Koole D, Zandvoort HJ, Schoneveld A, Vink A, Vos JA, van den Hoogen LL, et al Intraluminal abdominal aortic aneurysm thrombus is associated with disruption of wall integrity. J Vasc Surg. 2013;57:77–83. 10.1016/j.jvs.2012.07.003 23127983

[14] Grimes DA, Schulz KF. Compared to what? Finding controls for case-control studies. Lancet. 2005;365:1429–33. 15836892 10.1016/S0140-6736(05)66379-9

[15] Faul F, Erdfelder E, Buchner A, Lang A. Statistical power analyses using G*Power 3.1: Tests for correlation and regression analyses. Behavior Research Methods. 2009;41:1149–1160. 10.3758/BRM.41.4.1149 19897823

[16] Anaconda^™^ AAA Stent Graft System [Internet]. Renfrewshire, Scotland: Vascutek. Last opened: 28th of 12, 2014 Available: http://www.vascutek.com/Downloads/IFUs/Anaconda/Anaconda%20301-105_5.pdf#page=5.

[17] Zenith Flex: Planning and sizing [Internet]. Bloomington, IN:Cook medical. Last opened: 28th of 8, 2014 Available: https://www.cookmedical.com/data/resources/generalMarketingMatl/AI-BM-FXSZPSPPT-EN-201104.pdf.

[18] Franks SC, Sutton AJ, Bown MJ, Sayers RD. Systematic review and meta-analysis of 12 years of endovascular abdominal aortic aneurysm repair. Eur J Vasc Endovasc Surg. 2007;33:154–71. 17166748 10.1016/j.ejvs.2006.10.017

[19] White GH, Yu W, May J, Chaufour X, Stephen MS. Endoleak as a complication of endoluminal grafting of abdominal aortic aneurysms: classification, incidence, diagnosis, and management. J Endovasc Surg. 1997;4:152–68. 9185003 10.1177/152660289700400207

[20] Hobo R, Kievit J, Leurs LJ, Buth J, EUROSTAR collaborators. Influence of severe infrarenal aortic neck angulation on complications at the proximal neck following endovascular AAA repair: a EUROSTAR study. J Endovasc Ther. 2007;14:1–11. 17291144 10.1583/06-1914.1

[21] van Marrewijk C, Buth J, Harris PL, Norgren L, Nevelsteen A, Wyatt MG. Significance of endoleaks after endovascular repair of abdominal aortic aneurysms: The EUROSTAR experience. J Vasc Surg. 2002;35:461–73. 11877693 10.1067/mva.2002.118823

[22] Jilaihawi H, Kashif M, Fontana G, Furugen A, Shiota T, Friede G, et al Cross-sectional computed tomographic assessment improves accuracy of aortic annular sizing for transcatheter aortic valve replacement and reduces the incidence of paravalvular aortic regurgitation. J Am Coll Cardiol. 2012;59:1275–86. 10.1016/j.jacc.2011.11.045 22365424

[23] Schultz CJ, Weustink A, Piazza N, Tzikas A, Otten A, Nuis RJ, et al Three dimensional evaluation of the aortic annulus using multislice computer tomography: are manufacturer's guidelines for sizing for percutaneous aortic valve replacement helpful? Eur Heart J. 2010;31:849–56. 10.1093/eurheartj/ehp534 19995874

[24] Kaladji A, Lucas A, Kervio G, Haigron P, Cardon A. Sizing for endovascular aneurysm repair: clinical evaluation of a new automated three-dimensional software. Ann Vasc Surg. 2010;24:912–20. 10.1016/j.avsg.2010.03.018 20831992 PMC3001481

